# Otomyasthenia—Muscle-Deafness*Ear-Muscle Debility.

**Published:** 1898-03

**Authors:** Thomas F. Rumbold

**Affiliations:** St. Louis, Mo.


### OTOMYASTHENIA—MUSCLE-DEAFNESS.*

By THOS. F. RUMBOLD, M.D.,

ST. LOUIS, MO.

In the September number of the *Laryngoscope* I gave my views concerning the functions of the tensor tympani and stapedius muscles,† stating that these functions were to select and amplify such sounds as the listener desired to hear most distinctly. I have received some letters since the publication of this article asking me to give the clinical application of the deductions contained in the full paper.

In answer I will say, in part, that with a knowledge of the functions of these ear-muscles we are able to name, with a great deal of certainty, two causes of deafness that are not generally known. One of these causes is due to a paralysis agitans of these muscles, described at some length in the paper above mentioned; and the other cause of deafness is a debilitated condition of these ear-muscles, which prevents them from selecting and amplifying sounds normally, an *otomyasthenia*. The fact that asthenia of the ear-muscle or muscles is a cause of deafness is intimated in the

---

* Ear-Muscle Debility.

† The article was read at the Western Ophthalmological, Otological, Laryngological and Rhinological Association, April, 1897, and published in the Transactions.

full paper. In this paper I will add enough to enable one to easily select his cases of middle-ear muscle asthenia.

This disability is not very difficult of diagnosis; in fact the patient himself will almost invariably indicate the cause by the description of his deafness, as is plainly shown by the following from an intelligent patient, aged fifty-eight years. He says:

"I have no difficulty in understanding you, or even in hearing my little grandson, three years old, when he is talking to me from the head of the stairs, but some persons talk in such a mumbling way that their words are hard to comprehend. When I am on Change I can't make out one word, and I have great difficulty on the cars. If persons would speak plainly I could hear them very well. I can prove to you that I am not very deaf. When I go to a lecture, as soon as all are seated and the speaker gets started so as to stop all whispering, I hear him very well while I am fifty feet away from the platform. Last evening I was at a whist party; before the play commenced I could scarcely make out one word, because of the confusion made by all of them (sixteen persons) talking and laughing together. How they understood one another I don't know. As soon as quiet was restored I heard those at the other end of the room who once in a while made a remark to some one at their table, and the room was at least twenty feet long. You see I am not very deaf, but only so under certain circumstances. There is another curious thing; while I am eating I can't distinctly hear those on the other side of the table; the noise made in my mouth while I am chewing my food is so great as to drown the words, so I have to stop eating to hear them."

In the light of what was said in the paper above referred to, it will appear plainly that his deafness is due to the inability of his ear-muscle or muscles to select and amplify the sounds that he desires to hear, this disability being, in him, almost if not quite total; indeed much greater than is usually observed in this class of cases.

It should be noticed that it is only while other noises are being made that he complains of the mumbling way in which others talk. If the other noises were not present, his hearing, while it is not good, is such that he would not for an instant think of complaining. If a person with normal ears listens to the conversation of a friend, the words of others near him seem to be spoken in a mumbling way. In this he resembles exactly the disability of the man suffering from otomyasthenic deafness, for the simple reason that his ear-muscle or muscles are not endeavoring to select and amplify the sounds of the other persons. But if this listener will turn his attention to what one of the other persons is saying, that is, selecting that one's words instead of his friends, then instantly the words of his friend will seem as though spoken in a mumbling way. This is the daily experience of every person with normal ears.

When there was no necessity for selection and amplification, such as in the case of his grandson's voice, and in the quiet lecture room, he heard with entire satisfaction, but when he desired to hear certain selected sounds, as the words of one of the company in a roomful of noisy people, and not be bothered with the undesired sounds, his ear-organs were unable to perform the task. In a quiet room no one would consider this man very deaf. His statements of his ability to hear when there was no noise are ample demonstrations of this.

It is evident that this variable condition of his hearing is not due to an abnormal condition of the auditory nerve, for this nerve cannot be obtuse in a noisy room and then acute the instant the noise ceases. For the same reason it cannot be due to tinniti of either kind. Cases of this kind very seldom suffer from muscular tinnitus. If they do, it is very weak in intensity, a significant fact. Vascular tinnitus, in varying degrees of severity, is almost always present in these cases, but it can, obviously, cut no figure in causing variableness of the hearing. This proves that this condition of the hearing is due alone to the inability of the ear-muscle or muscles to select and amplify the desired sounds, a myasthenia.

There is another method of proving that asthenia of the middle ear-muscles is the cause of deafness than by that of the patient's history of his subjective symptoms; this is by the employment of the tick of the watch.* These patients frequently surprise the

*The louder the tick of the watch the more satisfactory the examination. A low tick shows so little difference that mistakes may be and are almost certain to be made, which will not occur with a loud ticker.

physician by the distance they can hear the metallic tick of the watch in a quiet room. This man heard the watch 20/96 R. 14/96 L. It varied a very little each time in the four or five tests that were made at his first visit. After getting his hearing by slowly bringing the watch *up* to his ear until he heard it, I then slowly carried it away from his ear to ascertain if he could hear it some distance farther away, but he *could not* do so with either ear, even after quite a number of trials. This, I consider, proves conclusively that he is afflicted with complete otomyasthenia.

Many persons who could not hear the watch at the distance he did, namely 20/96 R. 14/96 L., can hear the ordinary conversation of a person standing alongside of them with ease in a roomful of laughing and talking people, for the reason that their ear-muscles select and amplify the words of the person they desire to hear. This my patient could not do, because of the asthenic condition of his ear-muscles.

In every person with normal ears, and in all who are only partially otomyasthenic, the tick of the watch may be heard *farther* than it is heard when it is slowly brought *up* to the ear; that is to say, if he hears it when slowly brought up to the ear at twenty-four inches (which occurred in a partially otomyasthenic patient), the watch may be slowly taken *away* from the ear, and he may continue to hear it as far as to 30 or 36 inches, if the ear-muscles are not wearied by too long a test. It seems conclusive that the increased hearing distance demonstrates that his ear-muscles amplified the sound of the watch's tick, or he would not have heard it beyond the first hearing distance, twenty-four inches.

When these ear-muscle or muscles are in a *complete* asthenic condition, the will of the listener has lost the normal control over them; but, says one, these persons can hear; yes, but they are deprived of this extra acuteness of hearing, especially in a noisy place where the election of sounds they desire to hear most plainly is denied them, which, with normal ears, all persons enjoy. The proof of this is the hearing-distance of the watch cannot be increased even by an inch beyond the first hearing distance, it makes no difference how frequent the trials, or how slowly the watch is removed from the ear, or how much the listener exerts himself to hear it.

After daily examinations of the hearing-distance in this manner during the last five or six years, with a special view to this subject, I find that otomyasthenia, in varying degrees, is by far the most frequent cause of deafness, showing the importance of understanding its mechanism.

The following is a good method of making a differential diagnosis of this kind of deafness:

While in San Francisco this summer I chanced to step into a place where the Edison phonographs were on exhibition. I was accompanied by a physician who was under treatment for complete otomyasthenic deafness. Three of the machines were so arranged that the tubes led to one person's ears. One machine played Old Hundred, another Yankee Doodle, the third Annie Laurie. When my friend placed the ear-pieces in his ears, he heard a confusion of noises. The exhibitor said, "Listen and you will hear Annie Laurie;" but he could not. "Well, can't you hear Yankee Doodle?" He could not. "Old Hundred is there too." "No, sir, nothing but a confusion of noises that would drive one crazy if he listened to it very long." He could not hear any two tunes together, but instantly called out each of the three tunes as they were played singly. I then listened to the three machines, but could barely hear Yankee Doodle; when he stopped this machine I then heard Annie Laurie very well, when this machine stopped I heard Old Hundred, of course, proving plainly that I also was affected with otomyasthenia to a considerable degree. I took the ear-piece out of my right ear, and easily selected each tune with my left ear while all three machines were playing. I suffered a serious injury of my right ear in 1869, which rendered me quite deaf in this ear. This accounted for my disability.

Incidentally, I will say that the subjective symptoms of otomyasthenic deafness prove that my views concerning the functions of the middle ear-muscles are correct, namely: that their office is to select and amplify such sounds as the listener desires to hear most distinctly, showing that the ears have muscles of accommodation quite analogous to those of the eyes.

